# Women in mechanical circulatory support: She persisted!

**DOI:** 10.3389/fcvm.2022.961404

**Published:** 2022-10-13

**Authors:** Kelley N. Benck, Fatima A. Khan, Mrudula R. Munagala

**Affiliations:** ^1^Division of Cardiology, University of Miami Miller School of Medicine, Miami, FL, United States; ^2^Department of Cardiology, University of Texas Medical Branch, Galveston, TX, United States

**Keywords:** mechanical circulatory support (MCS), women in MCS, women in medicine, left ventricular assist device (LVAD), heart failure, CT surgeons, cardiologist

## Abstract

Many women physicians have blazed trails and played instrumental roles in advancing the field of Advanced Heart Failure (AHF), Mechanical Circulatory Support (MCS), and cardiac transplantation to its current recognition and glory. In contrast to other areas of cardiology, women have played an integral role in the evolution and emergence of this sub-specialty. Although the ceiling had been broken much later for women cardiothoracic (CT) surgeons in the field of AHF, the ingress of women into surgical fields particularly CT surgery was stonewalled due to pervasive stereotyping. The constancy, commitment, and contributions of women to the field of AHF and MCS cannot be minimized in bringing this field to the forefront of innovation both from technological aspect as well as in redesigning of healthcare delivery models. Integrated team-based approach is a necessity for the optimal care of MCS patients and forced institutions to develop this approach when patients with durable left ventricular assist devices (LVAD) began discharging from the hospitals to local communities. Women in various roles in this field played a pivotal role in developing and designing patient centered care and coordination of care in a multidisciplinary manner. While embracing the challenges and turning them to opportunities, establishing partnerships and finding solutions with expectations to egalitarianism, women in this field continue to push boundaries and subscribe to the continued evolution of the field of AHF and advanced cardiac therapies.

## Introduction

**FIGURE 1:**
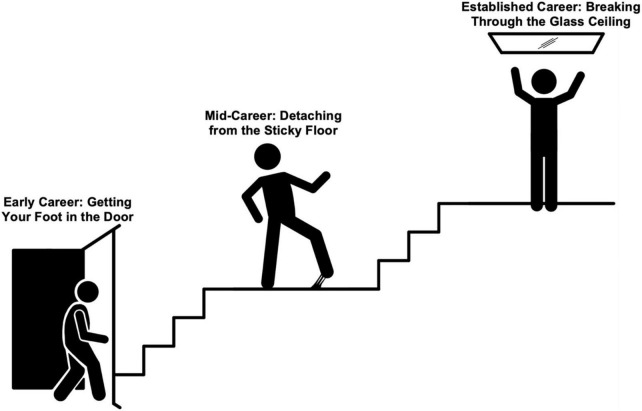
Stages of challenges facing women entering heart failure and CT surgery.

In the traditionally male dominated profession of medicine, addressing gender disparities continues to spark passionate debate. Despite ongoing efforts and closing the gender gap among medical school matriculants, women have yet to achieve equality in multiple disciplines of medicine and surgery. In the United States, approximately one in four trainees in cardiology and thoracic surgery is a woman, reflecting a significant gender gap in these specialties (1). The exception is seen in pediatric cardiology, where just over half of fellows have been women. The reasons for consistent underrepresentation of women in adult cardiology and cardiothoracic (CT) surgery fields are myriad. Women in these fields experience difficulties at multiple levels including (1) gaining entry (unlocking the door), (2) barriers to promotion, impediments to professional development, and delayed or limited career advancement (sticky floor), and (3) lack of leadership opportunities among institutions, professional societies, and editorial boards (glass ceiling) as represented in Figure 1. In spite of these challenges, women in these specialties have marched forward and redefined the fields with their professionalism, contributions and commitment, and constancy.

Evolution of AHF, MCS and transplant took a huge leap with the approval of LVAD implantation as a destination therapy (DT) and has transformed over the years from a primarily a surgically driven model to a multidisciplinary model due to the complexities involved in the care of these patients. As a lot of initial work in the field of MCS and transplantation was driven by CT surgeons, the role of women in the early stages of the field was paltry as the field of CT surgery was considered to be a man’s field. Dr. Nina Starr Braunwald became the first woman to perform CT surgery in the United States breaking the sociocultural barriers and the pervasive stereotyping (2). It is the perseverance of pioneering women surgeons such as Drs. Nina Braunwald, Ann McKiel, and Nermin Tutunju that paved the path for women of future generations (3). These women blazed a trail and made CT surgery an achievable aspiration for other women who subsequently followed in their footsteps and in later years opened the window to surgical management of AHF including MCS and cardiac transplantation.

After the Randomized Evaluation of Mechanical Assistance for The Treatment of Congestive Heart Failure (REMATCH) clinical trial helped to establish LVAD implantation as a viable therapeutic option for patients with end-stage heart failure who are not candidates for cardiac transplantation, the role of heart failure cardiologists rose to prominence in the care of these patients and forced the evolution of this field as a distinct specialty (4). Conventional healthcare delivery models were challenged with LVAD patients living in the communities as the care of these patients required care coordination and multidisciplinary team (MDT) approach. The MDT care model of heart failure and MCS patients has been adapted by various medical and surgical specialties and has evolved into a standard of practice currently for patients with complex care needs.

While gender equity and parity have yet to be fully attained in this field, tremendous progress has nonetheless been made in this field as compared to other subspecialties of cardiology. Similar strides have been made in CT surgery over the past decade to reduce the gender disparity and recruit women through mentoring, advocacy, and sponsorship (5). In this review, we focus on the experiences and views of women cardiologists and CT surgeons in the field of MCS regarding the perceived barriers, challenges, growth opportunities and future directions for the women in this field.

## Methods

Although this is a review, we followed an integrated approach and combined the information obtained through literature review and shared experiences from interviewees in presenting this article. For this review, we interviewed women in both current and past leadership positions in this industry. We sought to understand their motivations, challenges, and achievements to highlight the path of women in these fields over multiple generations. The interviewees included in this review were by no means intended to be an exhaustive list. We broadly categorized the interviewee’s experiences into medical [advanced heart failure (AHF) cardiologists] and surgical (CT surgeons) perspectives based on the respective area of their professional practice. In addition, we reviewed the literature on women physicians in cardiology and, cardiothoracic surgery, medicine, and in academic medicine. We integrated the information derived from literature review with information shared by our interviewees due to the specific focus on the field of MCS. Although it is imperceptible to cleave AHF, MCS and transplantation, this article kept the primary focus on the role of women cardiologists and cardiothoracic surgeons in the field of MCS. We hope to illuminate the path and perseverance of these women to acknowledge their grit and dedication, as well as to provide a framework of success for future women of these fields. We have listed the Heart Failure Cardiologists and CT surgeons we spoke to with their current titles and leadership positions in alphabetical order as represented in Table 1.

**TABLE 1:** List of interviewees for this review and their current affiliated institutions.

Name	Current institution	Area of expertise (practice)
Deborah Ascheim, MD	StrideBio, Inc.	AHF, MCS, and Transplant Cardiology
Geetha Bhat, MD	Penn State heart and Vascular Institute, Hershey, PA, United States	AHF, MCS, and Transplant Cardiology
Linda Bogar, MD	AdventHealth Transplant Institute, Orlando, FL, United States	CT surgery
Elizabeth Blume, MD	Boston Children’s Hospital, Boston, MA, United States	Pediatric AHF, MCS, and Transplant Cardiology
Susan Brozena, MD	University of Pennsylvania Health System, Radnor, PA, United States (Retired)	AHF, MCS, and Transplant Cardiology
Margarita Camacho, MD	Newark Beth Israel Medical Center, Newark, NJ, United States	CT surgery
Jennifer Cook, MD	University of Cincinnati, Cincinnati, OH, United States	AHF, MCS, and Transplant Cardiology
Hannah Copeland, MD	Lutheran Hospital- Fort Wayne, IN, United States	CT surgery
Jennifer Cowger, MD, MS	Henry Ford Health, Detroit, MI, United States	AHF, MCS, and Transplant Cardiology
Amy Fiedler, MD	University of California San Francisco, San Francisco, CA, United States	CT surgery
Maya Guglin, MD, Ph.D.	University of Indiana Health, Indianapolis, IN, United States	AHF, MCS, and Transplant Cardiology
Amy Hackmann, MD	University of Texas Southwestern Medical Center, Dallas, TX, United States	CT surgery
Shelley Hall, MD	Baylor University Medical Center, Dallas, TX, United States	AHF, MCS, and Transplant Cardiology
Sharon Hunt, MD	Stanford University Medical Center, Stanford, CA, United States	AHF, MCS, and Transplant Cardiology
Mariell Jessup, MD	American Heart Association	AHF, MCS, and Transplant Cardiology
Manreet Kanwar, MD	Allegheny General Hospital, Pittsburgh, PA, United States	AHF, MCS, and Transplant Cardiology
Anuradha Lala-Trindade, MD	Icahn School of Medicine at Mount Sinai, New York, NY, United States	AHF, MCS, and Transplant Cardiology
Sharon Larson, DO, MS	University of Iowa, Iowa City, IA, United States	CT surgery
Seema Mital, MD	SickKids Research Institute, Toronto, Ontario, Canada	AHF, MCS, and Transplant Cardiology
Stephanie Moore, MD	University of Louisville, Louisville, KY, United States	AHF, MCS, and Transplant Cardiology
Salpy Pamboukian, MD	University of Alabama at Birmingham, Birmingham, AL, United States	AHF, MCS, and Transplant Cardiology
Brigitte Stiller, MD	University of Freiburg, Breisgau, Germany	AHF, MCS, and Transplant Cardiology
Nancy Sweitzer, MD, Ph.D.	Washington University School of Medicine, St. Louis, MO, United States	AHF, MCS, and Transplant Cardiology

## Pioneers of advanced heart failure and mechanical circulatory support field

Over the past three decades, the emergence of the LVAD as a durable therapy for end stage heart failure revolutionized the field of AHF and MCS. This has transformed the operational framework of the field from a primarily surgeon driven model to a multidisciplinary team approach. This novel archetype brought the need for a new breed of cardiologists to facilitate the longitudinal care of patients with devices. Women cardiologists who found this specialty attractive due to complexity of the patients collaborated with surgeons, engineers, and industry, which enabled them to develop partnerships, a fundamental key to building successful AHF and MCS programs and services. They established programs with their remarkable leadership, and team building skills and played instrumental roles in the evolution of this field. Noteworthy leadership in this field includes Dr. Sharon Ann Hunt, Dr. Hannah Valantine, Dr. Mariell Jessup, Dr. Lynne Warner Stevenson, Dr. Maryl Johnson, Dr. JoAnn Lindenfeld, Dr. Maria Generosa Crespo-Leiro, Dr. Donna M. Mancini, Dr. Maria Rosa Costanzo, Dr. Anne M Keogh, Dr. Heather Joan Ross, Dr. Geetha Bhat, Dr. Ileana Piña, Dr. Mary Norine Walsh, Dr. Roberta C. Bogaev, Dr. Nancy K Sweitzer, Dr. Emma Jane Birks, and Dr. Shelley Anne Hall. They have brought transformative changes that have shaped the field and defined the role of women in this field (6, 7).

Jean Rosensaft co-founded LVAD technology, Inc., in 1983 along with Dr. Adrian Kantrowitz and served as vice president of this bioelectronics research company that was focused on developing mechanical circulatory support (MCS) devices. A Japanese CT surgeon Dr. Chisato Nojiri joined the research team of Professor Willem Johan Kolff who was known as “father of artificial organs” at University of Utah after acquiring her Ph.D. in Japan. She focused on development of LVADs subsequently and developed a magnetically levitated implantable LVAD (8). It is the pathbreaking entry of Dr. Margarita Camacho in the clinical practice of AHF and MCS that has transformed the field for women CT surgeons. Brilliance, sedulity, and collective efforts of these women in various realms of the AHF and MCS propelled the field to its current distinguished status and pushed to create more space for women around them and for women of next generations.

## Finding a path to the heart

### Shaping the field

Dr. Sharon Ann Hunt is one of the originals in the field of AHF, MCS and Cardiac transplantation. She has witnessed the evolution of the field and informally addressed as “mother of transplant.” Although she focused more on the care of heart transplant patients, she was involved in the care of Robert St. Laurent, a patient who underwent the first successful implantation of LVAD as a bridge to transplantation with a Novacor device. She wrote commentary for the REMATCH study, a landmark clinical trial demonstrating the survival benefit of LVAD as a destination therapy. Dr. Marriell Jessup is a thought leader and a pioneer in the field of AHF and has served in many roles that positively impacted the field. The efforts of pioneering physicians such as Drs. Sharon Hunt and Mariell Jessup culminated in recognition of AHF as a secondary specialty of cardiology. Dr. Jessup played a crucial role in developing the curriculum and establishing the standards of training and competency requirements for the heart failure subspecialty. Dr. Lynne Warner Stevenson, recognized as doyenne of MCS field played a crucial role in designing the Interagency Registry for Mechanically Assisted Circulatory Support (INTERMACS) for monitoring of outcomes and in developing INTERMACS risk profiles to better characterize the clinical risk of AHF patients (9). Dr. Birks’s contributions to myocardial recovery of patients with LVAD are noteworthy (10). Dr. Hall organized the “Women in Transplantation and MCS” group and laid the foundation for collective action for advancement of women in this field. She played an instrumental role in bringing women in this field together and providing a platform for collaboration, support, and networking (11).

Dr. Chisato Nojiri, who dreamed of becoming a Nobel laureate in physics and later became a physician scientist, was deeply invested in the development of durable MCS devices. Later, she became the CEO of Terumo Heart Co., Ltd., and was successful in executing the development and commercialization of magnetically levitated implantable LVAD (DuraHeart) (12). The first woman cardiothoracic surgeon to implant a durable LVAD in clinical practice in the United States (presumably in the world) to our knowledge is Dr. Margarita Camacho. She opened the door for other women in the CT surgery field to join this very rewarding AHF and MCS realm of CT surgery. There are several female CT surgeons in the field currently and we compiled a list based on online search in alphabetical order, Table 2. The excitement, gratification, and cachet of offering a new life to someone with end stage heart failure and its impact on the patients and their families are the driving forces for women to enter the surgical specialty of AHF and MCS.

**TABLE 2:** List of female CT surgeons currently in the field of MCS (to our knowledge based on internet search).

CT surgeons in MCS field	Current affiliated institution
Linda Bogar, MD	AdventHealth Transplant Institute
Reshma Biniwale, MD	University of California Los Angeles
Margarita Camacho, MD	Newark Beth Israel Medical Center
Hannah Copeland, MD	Lutheran Hospital- Fort Wayne, IN
Amy Fiedler, MD	University of California San Francisco
Amy Hackmann, MD	University of Texas Southwestern
Sharon Larson, DO, MS	University of Iowa
Marzia Leacche, MD	Spectrum Health
Deyanira Prastein, MD	Rochester Regional Health
Alexandra Tuluca, MD	Einstein Healthcare Network
Katherine Wood, MD	University of Rochester

This section will delve into what inspired and persuaded the women we interviewed to join these teams and purse these fields. We interviewed physicians of different generations who are at different stages in their professional development with varied career trajectories including physicians in clinical practice, academic practice, and physician scientists. While the earlier generation physicians had latitude to design and shape the field, later generations of interviewees touted their fortune to have many pioneering female physicians as their role models. Below we intend to highlight some of these trailblazing women whose work were inspiring to many of our interviewees. Comparable to this review, the list above by no means holds all female physicians to inspire and impact the field. These names represent some of the profound work that have progressed the practice of medicine for patients with AHF. Now having acknowledged some of these historical efforts, the remainder of this section will highlight themes that arose in our interviews with heart failure specialists and CT surgeons.

### Perspectives of heart failure cardiologists

Few of the physicians we interviewed were successive generations of physicians or came from a medical background, but many had a passion to become a physician during their childhood. As heart failure was a relatively new branch of cardiology and a niche specialization, an interest in pursuing that fellowship came much later for most of the interviewees. Heart failure patients have chronic health needs facilitating longitudinal care allowing to build lifelong relationships with them. Dr. Mariell Jessup, Chief Science and Medical Officer of the American Heart Association and an Emeritus Professor of Medicine at the University of Pennsylvania School of Medicine, who is instrumental in carving out heart failure as a subspecialty stated that she derives motivation primarily from patients and patient stories (13). One physician informed us her drive to serve this population comes from the unique needs of care that this population has and shared her experiences of having to act as primary care physician for these complex patients. Where there are moments of immediate gratification when there is a noticeable clinical difference with institution of a temporary MCS device, there are other instances that demand patience and time to know the trajectory of clinical course and outcome. As the mortality in AHF is high, the field requires an ability to have end of life and advanced life care planning conversations with a patient’s loved ones, a responsibility many of the women we interviewed held dearly.

Despite perceived limitations and challenges, heart failure as a specialty has been a rewarding experience to the physicians we spoke with. Given the high acuity in end stage heart failure, patient outcomes are not always favorable. Workdays are long and patient needs may arise at any hour of the day or night leading to grueling work hours and burnout. Though not all these challenges are unique to heart failure, there has been a decline in fellowship applicants to heart failure and transplant programs. In 2021, there were twice as many fellowship applications for interventional cardiology as for heart failure (2, 14). We inquired about this difference and our interviewees attributed this diminishing interest to multiple factors including the predominant nature of inpatient experience of heart failure during fellowship training, current model of pay structure based on relative value unit (RVU) billing system that is more lucrative for procedure-oriented cardiology secondary specialties in comparison to heart failure and perceptions of less autonomy in decision making due to MDT approach. Many women acknowledged the specialty had more delayed gratification compared to some other cardiology specialties and current models of value analysis and reimbursement do not effectively capture the efforts of heart failure cardiologists and the cognitive input required in taking care of these complex patients. In addition, heart failure cardiologists carry a significant emotional burden as the mortality of heart failure patients is higher than most cancers leading to channeling a portion of practice into end-of-life discussions.

Overall, there was an acknowledgment that no field is perfect. However, so many of our interviewees felt a strong calling to do such demanding work. They felt the reward of their career from the genuine passion they held to serve such unique heart failure patients.

### Perspectives of cardiothoracic surgeons

Akin to the heart failure specialists we interviewed, many of the CT surgeons we spoke with did not come from generations of physicians but found a passion for surgery earlier in life. Most of the interviewees were motivated to become surgeons well before finding cardiac surgery, which then led them to MCS and transplant. Passion for this subspecialty partly came from a fascination with the heart itself. Some described the neatness of the heart being one of the few organs that we can physically feel functioning, while others were fascinated by the physiology of the heart. Comparable to other fields of surgery, there is an immediateness to the results of their work, but transplant and MCS are unique as these are some of few surgical subspecialties that involve putting something into the body rather than taking out. Many of the surgeons we spoke to enjoy the team-based approach used within MCS and transplant, and for the opportunity to collaborate with other specialties.

Great progress has been made in the technology and techniques of MCS and transplant and the surgeons we spoke with were excited about the accelerating pace of changes in the field and had varying opinions of the future path of MCS and transplant. Enthusiasm is on the upswing in the sphere of transplant as xenotransplantation has recently made headlines and donation after cardiac death (DCD) continues to expand into more institutions. The frontiers of MCS are being pushed to achieve better hemocompatibility, miniaturization, and thermal energy transfer systems with hope of developing a fully implantable LAVD free of external cables. There is boundless excitement regarding the potential for application of artificial intelligence and machine learning in the clinical care of these complex patients and shift the paradigm towards precision medicine. Some felt that success of HeartMate 3 LVAD carved a future with greater use of MCS, while others felt the expansion of the donor pool and transplant techniques including xenotransplantation may open more avenues in the transplant arena. Regardless of the specialty’s direction, some aspects are unlikely to change. Transplant cases can arise at any hour of the day and require significant coordination from procurement to transplantation leading to long and arduous days, presenting challenges in many surgeons’ personal lives.

Though the work as a CT surgeon in MCS and transplant may be challenging, it was evident that the women we spoke with held genuine admiration for the field and enjoyment of the work they were performing. As one surgeon said, it is not for the faint of heart, but if you love it, go for it.

## Sponsorship is the new mentorship

### Perspectives of heart failure cardiologists

All women cardiologists we spoke to acknowledged the power and influence of mentorship in their professional development. While no one denied the significance of having a mentor that looks like you, the preference is unanimous among the interviewees for a quality mentorship over gender concordance. Many of the women we interviewed had predominantly male or only male mentors, often due to a lack of female leadership during the time of their training. They felt being seen for their work provided the greatest value as well as having mentors that brought them to the table. One example of this is seen with Dr. Elizabeth (Betsy) Blume current Director, Advanced Cardiac Therapies; Medical Director, Heart Failure Program at Boston Children’s Hospital. Earlier in her career she was mentored by and collaborated with both cardiologist and surgeons who brought her into a multi-institutional study based on Pediatric Heart Transplant database that analyzed the outcomes of children bridged to heart transplantation with ventricular assist devices (15). Their collaboration continued and she played a major role in establishing Pediatric Interagency Registry for Mechanical Circulatory Support (PediMACS) (16). This inclusion and acknowledgment of her abilities allowed her a steppingstone for many future collaborations and leadership opportunities. Dr. Blume’s story illuminates that quality mentorship does not only include setting an example but bringing mentees into the conversation and allowing them opportunities for authorship and leadership.

Expanding the role of mentorship into one of sponsorship brings inclusion and promotes their protégé. This idea was proffered by Dr. Salpy Pamboukian—Director, MCS Device Program; Co-section Head, Advanced Heart Failure, Cardiac Transplantation, MCS, and Pulmonary Vascular Disease at University of Alabama at Birmingham, while describing her own experiences as both a mentee and a mentor. The most transformative and powerful relationships were reaped when a mentor took an active interest in her career, “shepherding” her toward various opportunities. Many of the women spoke of the reciprocal relationship they cultivated as mentees and sought for as mentors.

### Perspectives of cardiothoracic surgeons

The cardiothoracic surgeons we interviewed similarly felt that the quality of the mentorship was more impactful than the gender of the mentor. Two common themes we found that made mentorship impactful were belief and inclusion. Each interviewee described a unique experience of having to balance support and discouragement on their path from first deciding to pursue a career in medicine to training as a cardiothoracic surgeon. Regardless of any negativity they may have faced, their self-confidence, grit, and dedication helped in establishing quality mentor relationships. Though the poise previously described was not unique to any one surgeon we spoke to, Dr. Linda Bogar is one example of perseverance manifesting mentorship. She is a CT surgeon at AdventHealth Transplant Institute, Orlando, FL with a focus in heart and lung transplant and MCS, and previously served as the Surgical Director of Heart Transplantation and MCS at Thomas Jefferson University Hospital. Dr. Bogar found her calling to CT surgery late in the fourth year of her general surgery residency. Since the realization came after the deadline for the CT fellowship match, her chief at the time facilitated a 1-year “fellowship” as a substitute to prepare her for her actual CT fellowship the following year. This story highlights the reciprocal relationship seen in quality mentorship. Seeing and understanding Dr. Bogar’s hard work and dedication, her mentor supported and believed in her to aid in her success. Many women that we interviewed felt that the investment in time and energy from their mentors came partly from a place of reciprocal respect. Belief in one’s own capabilities and hard work created an environment for their mentor to both believe in them and put effort into their success. Every woman had at least one male mentor in their life invested in their growth and navigated supported opportunities for them. Most women of recent generations described being lucky enough to have at least one female mentor. Dr. Amy Fiedler, Assistant Professor of Cardiac Surgery at The University of California, San Francisco and member of the Presidential Leadership Scholars Program, discussed one such example of this female mentorship. Dr. Fiedler described the influence of cardiac surgeon, Dr. Jen Walker, on her journey from being a general surgery resident to finding CT surgery. Dr. Walker not only encouraged Dr. Fiedler, but also made her realize that there was a space for someone like her as a woman in cardiac surgery. Though the sponsorship provided to Dr. Fiedler was impactful on her career, she posited that inclusivity and representation are crucial for success of the women entering such male dominated fields of medicine such as cardiology and CT surgery. Dr. Pamboukian summed up this concept in her statement “the ability to have a quality mentorship should not be dependent on race, ethnicity, age, or gender.”

## Wearing one hat at a time: Work-life balance

### Perspectives of heart failure cardiologists

The most common response when asked about work-life balance is that no such thing exists. Many of the women we interviewed discussed the need to prioritize the different aspects of life at different times. This idea was well illustrated by Dr. Anuradha Lala-Trindade (Anu Lala), Director of Heart Failure Research, who serves in a leadership role at the Data Coordinating Center for the NHLBI CT Surgery Network, director of the fellowship program in AHF and Transplant at Mount Sinai, and a Deputy Editor at the *Journal of Cardiac Failure*. She described being moved by the notion that you can do it all, just not at the same time. Feeling one should be able to “wear all these different hats at all the same time” and expecting to excel at all one’s different roles, is setting oneself up for frustration. Many of these women learned to focus on the different aspects of their life at different days and different times.

This prioritizing of responsibilities was echoed by Dr. Shelley Hall—current Chief of Transplant Cardiology, MCS and Heart Failure at Baylor University Medical center, President of Texas American College of Cardiology (ACC) and founded the Women in Transplant and MCS organization. She is the past Chair of UNOS cardiac committee and Thoracic and Critical Care Council of Practice, American Society of Transplantation (AST). She described how she often was unable to be as social at work as some of her male colleagues because each day she had to fulfill her duties as a physician before going home to her second job, motherhood. Dr. Hall and many other women we spoke to described the guilt that came with having a rigorous career as a heart failure cardiologist while being a mother. Some days they would be able to be a “great mom,” being home for dinner, participating in school field trips, or baking cookies for class activities, while other days they would be a “great physician” by taking extra-call or completing research goals. Though they never failed to fulfill their duties as a care provider of AHF patients, many felt their gender posed unique challenges.

Seeking this work-life integration was also discussed by Dr. Deborah Ascheim, current Chief Medical Officer of StrideBio, Inc., and former Director of the International Center for Health Outcomes and Innovation Research’s Clinical Trial Unit at the Icahn School of Medicine at Mount Sinai. Dr. Ascheim described her passion for working with critically ill heart failure patients in the ICU; however, the demands of the clinical practice combined with her clinical research activities were not always compatible with her own needs as a mother. Therefore, for a couple of years earlier in her career while serving as a heart failure cardiologist at Columbia University Medical Center, she carved her job in an albeit limited fashion to accommodate her personal needs even when such molding was not totally in line with her immediate professional goals. Dr. Ascheim felt this compromise may have sustained her longer in the field and opened future opportunities for her. She advised that women utilize negotiation skills to not only promote what they can offer their employer, but also to push and see what their employer can offer them. This synergy in the various hats these physicians wear, personally and professionally, are crucial for optimization of their abilities and overall wellbeing.

### Perspectives of cardiothoracic surgeons

The cardiothoracic surgeons we interviewed preferred to rename work-life balance to work-life “integration.” As one of the surgeons stated, “you have to take life one day at a time.” Similar to the experiences of the heart failure cardiologists, the female surgeons we interviewed felt it is unrealistic and unhealthy to take on the many roles of professional and personal life simultaneously. One piece of advice that was offered to help with this integration was practicing and executing strong time management skills. One example of this was described by Dr. Hannah Copeland, current Surgical Director of Heart Transplant and MCS and Director of ECMO at Lutheran Hospital in Fort Wayne, Indiana. She would design her days to ensure a distinct presence in each of her roles as a mother, as a wife, and as a surgeon. Each morning she would wake-up at 3:00 AM to perform chart review, read literature, or complete other work tasks and workout, before her kids would wake up. She designated specific time for her professional and personal activities, and crafted her routine to allow her to be fully present with her children after work until their bedtime, while fulfilling her professional interests. Though many specialties of medicine, especially surgery, are not foreign to early mornings and long days, this example demonstrates the kind of time management and alterations many of these women needed to make for their personal lives and loved ones.

An unfortunate aspect of work-life integration in CT surgery is the need for sacrifice. All of the surgeons we spoke to recalled the challenge of turning away from aspects of their personal life in order to adequately fulfill their duties and serve their patients. This can range from an inability to provide a reliable timeframe for family and friends to missing important family events. Though such sacrifice can at times be frustrating and difficult, in our interviews, we noticed an acceptance of what was required of them as surgeons without any regrets.

## To be or not to be: One of the boys

The occupational minority status of women in cardiology and thoracic surgery is well known, and the effect of this is felt differently by each woman. Overall, many seemed to identify a progression over the years from needing to be “one of the boys” in order to become successful to nowadays where many trainees want to be acknowledged as a woman doing the same work as men. This evolution of the perspectives and experiences of female physicians in these sub-specialties may be attributable to various factors including societal changes toward career women, greater discussion of representation and salary equality, and generational changes in work-life priorities.

### Perspectives of heart failure cardiologists

Earlier generations of physicians that we spoke to discussed how being one of the only women in their field was the *status quo*. One such woman is Dr. Sharon Hunt, one of the true pioneers of this subspecialty. As a fellow at Stanford University, Dr. Hunt and her three colleagues developed institutional guidelines for care of post cardiac-transplant patients (6). She served in various in leadership roles including Medical Director of the Heart Transplant Program at Stanford University and President of the International Society for Heart and Lung Transplantation (ISHLT) in 1996. She received several distinguished honors and awards including ISHLT Lifetime achievement award in 2012 and Hewlett award in 2013. She discussed the uniqueness of being one among the first to start treating post-cardiac transplant patients and the camaraderie she shared with her male colleagues. Being in an environment where energy was focused on the work being done and not the gender of the provider, created a space for her to be acknowledged as a capable, accomplished physician and not isolated by her gender.

Later generations of heart failure specialists encountered more gender-based stereotypes and shared experiences where their gender became more of a focus than their work. Overall, most physicians we interviewed were aware of underrepresentation of women leaders and role models in heart failure but did not use it as a deciding factor when choosing to enter the specialty. A few women we spoke to were motivated by the opportunity to close the gender gap; however, regardless of whether gender disparities were in the minds of these accomplished interviewees, everyone strongly felt that they should be recognized for their work independent of their gender. The diminished presence of women is not particularly limited to institutional and organizational leadership positions, but also evident at national conferences, industry sponsored events, editorial boards, and scientific societies. Dr. Nancy Sweitzer, Vice Chair of Clinical Research for the Department of Medicine at Washington University School of Medicine in St. Louis, and current editor-in-chief of *Circulation*: *Heart Failure* shared one such experience with us. She previously served as Chief of Cardiovascular Medicine and Director of the University of Arizona Sarver Heart Center. While attending a steering committee meeting for a heart failure device, she found herself to be the only woman on the committee and the only woman in the room that was not a member of the device company workforce. When she pointed this out to the members of the company, Dr. Sweitzer received a reply that they didn’t know any women, to which she responded by writing them an almost 40-person list that later became known as “Nancy’s List” within the company. In our interviews, we found a consensus that some of the modern issues with gender representation in heart failure came from a lack of trying to find women rather than overt exclusion. One’s career goals can be accomplished, but today’s culture may mean that those goals take longer to accomplish for women in this profession compared to their male colleagues. However, the goal for so many in the field can be best expressed by Dr. Sweitzer when she said she looks for the day where there are not men and women scientists, but just scientists.

### Perspectives of cardiothoracic surgeons

Gender inequity is abound in CT surgery training programs, practice and organized CT surgery and there is a dearth of female role models. Dr. Margarita Camacho shared with us that she was the only female site principal investigator on several of the ventricular assist device (VAD) clinical trials in the United States for many years as AHF surgical field was dominated by men. She is the current Surgical Director of the Cardiac Transplant and Mechanical Assist Device Program at Newark Beth Israel Medical Center and previously served as Chair of the Society of Thoracic Surgeons Workforce on End-stage Cardiopulmonary Disease and President of the Society of Women in Thoracic Surgery. Dr. Camacho is just one example of only a handful of trendsetting women that forged a path in an era with no or limited female leaders. When asked about the gender-specific challenges she faced, she described an experience similar to Dr. Hunt. She discussed how her success came from focusing on her work and outcomes, which was also the focus of her male colleagues. She was not isolated because of her gender but acknowledged as a capable surgeon.

Many physicians we spoke to from later generations discussed more gender-specific challenges in their career. Interestingly, almost all the women we spoke with described a mostly supportive environment during training but faced difficulties due to their gender when they entered into a faculty or staff position. These challenges ranged from micro-aggressions—such as being referred to by their first name at national conferences, while male colleagues in the same setting were referred to as “Dr. X”—to macro-aggressions like being told “why don’t you just get married” when asking for more time in the operating room. These attitudes were never easy to handle, but each of these physicians found different motivations to push through, such as an overriding passion for their work or a desire to fill the gender gap. One powerful piece of inspiration came from Dr. Sharon Larson, current Surgical Director of the Extracorporeal Membrane Oxygenation (ECMO) Program at the University of Iowa and the first female cardiothoracic surgeon to practice in the state of Iowa. She spoke about patient outcomes being her greatest motivation for her work. During Dr. Larson’s training she continuously strived to let her work speak for itself to focus the energy of her superiors on her role as a surgeon. If patients were being taken care of and skills were being developed, then “that is what [superiors] paid attention to and not necessarily the package from where the patient care and skill sets were coming from.” Throughout her training, she would constantly seek feedback, but there were times where her gender, rather than her work, were the focus of the feedback. Though she never stopped striving to improve her skills, she started incorporating the feedback from her patients as her guiding force. “You can’t be a physician without a patient.” Becoming the first female CT surgeon in Iowa and later taking on her directorship role did create some gender-specific challenges; however, the motivation provided by gracious patients, some over many years, continues to ignite her passion for CT surgery.

Regardless of how their gender impacted their career or interactions with colleagues, every physician—heart failure specialists and CT surgeons—we interviewed agreed they wanted their work to speak for itself. For the physicians that do not want to be part of the “boys club,” they do not desire special accommodations or privileges for their gender, but rather to be seen as equal for putting in the same work and having good patient outcomes. Though we saw a generational-based pattern in experiences, there surely exists differing intra-generational perspectives on gender. Some physicians we spoke to underscored the friendships and relationships they developed with their female colleagues during their careers within their institutions and across the organizations that fostered inclusion and branded themselves as “good girls clubs.” Many of our interviewees agreed with the notion of women to women support, but also advised a need for genuine passion for the work, belief in oneself, and grit.

## From being led to becoming a leader

### Perspectives of heart failure cardiologists

Though women were incremental in the formation of the heart failure specialty, it has taken time to achieve greater representation of female leaders. Current trainees have many accomplished women to emulate; however, there is still more work to be done. The current and past leaders we interviewed were often goal-driven in their pursuit and execution of their leadership. Though these positions were earned through merit, gender-specific obstacles and barriers that these women encountered cannot be disregarded. We would like to highlight Dr. Brigitte Stiller for her groundbreaking contributions to the Berlin Heart in pediatric cardiology including development of institutional protocols and she became the first female president of the German Society for Pediatric Cardiology. Dr. Stiller reminisced her experience of this role as first female president was initially daunting as it was a position held by men for over 50 years. However, when the first meeting with her as president came, she remembered that when the men before her spoke, everyone was quiet and listened, and she knew to expect nothing less while she led. It was her male colleagues’ responsibility to learn that there was a role change, and not for her to adapt to them. Dr. Stiller’s described female leadership was powerful and poignant and never disrespected. She encouraged academic debate and differing opinions—understanding their benefit and importance—but made sure to keep conversations on topic. The confidence she learned came from a multitude of life experiences, but also her time in the ICU helped develop her skills in collaboration.

As the field of transplant and MCS transitioned from surgery-derived care to heart failure specialists, an MDT approach evolved. Although there may be some institutional variations, MDTs expanded with time and our interviewees played an integral role guiding their team members and developing common purpose. Dr. Stephanie Moore is the current Medical Director of the AHF Therapies Program at the University of Louisville and previously served as Program Director for Massachusetts General Hospital AHF Fellowship program. She described a strategically adaptive leadership depending on the hat she wears. She predominantly followed a coach-style approach while guiding MDTs and a mixed approach (coach and democratic styles) with her fellows in training (FIT). Dr. Moore believes that maintaining a positive spirit in these leadership roles is essential as her team is feeding off her energy and relying on her expertise and guidance. Most physicians we interviewed felt a need to lead by example, which some described as “being in the trenches” with your team. When specifically speaking about training fellows, Dr. Moore and many other physicians equated the process to raising children. A gentle balance between supportive supervision and fostering autonomy is critical for the development of trainees to independently learn and grow from their mistakes in a safe environment. An essential element in leading a health care team or governing FIT is identifying a common goal by listening and understanding the perspectives of each team member and uniting the team around this common goal while aligning individual motivations to the common goal and nurturing individual needs and ambitions.

### Perspectives of cardiothoracic surgeons

Many of the leaders in cardiac surgery we interviewed were recruited to their roles due to their niche skill set or a forte that may serve the institutional goal and/or serve their community needs. Building trust is a key element to succeed in a leadership position. One surgeon we interviewed was Dr. Amy Hackmann, previous Surgical Director of Lung Transplantation, ECMO, and MCS at Keck Hospital of University of Southern California and current Surgical Director of the ECMO program at UT Southwestern Medical Center. She emphasized the importance of gaining trust to provide strong patient outcomes. One way Dr. Hackmann builds such rapport is by creating a culture of strong communication. Patients with AHF are complex and all possible routes of treatment must be considered. A thoughtful approach to these options with careful assessment of the ratio of risk versus benefits and partnership with patients and their caregivers are required in decision making. It is important to listen, talk through problems and allow academic debate. In addition, Dr. Hackmann has always been motivated by patient outcomes and allows her work to speak for itself. Finding this self-confidence is a requisite when assuming leadership positions for effective team building and earning the trust of the team in one’s competence and character is crucial for the success.

Complementary to trust building, these leaders of CT surgery also strive to create a culture of inclusivity. Bringing people to the table and increasing representation can strengthen the diversity of thinking and perspectives of an MDT. Representation is an important component of inclusivity, as well as attitudes. Many of these women highlighted this concept by discussing how they ensure to give credit when credit is due. Lastly, strong team culture comes from a leader understanding the individual attributes of each team player. Some of the surgeons discussed how they spent time understanding individuals’ strengths and weaknesses. Some people possess skills of empathy but lack efficiency of work or have strong productivity but are prone to oversight. Acknowledging these strengths and placing these individuals in complementary roles builds a stronger, more cohesive team.

## A path left behind

To leave behind a legacy is to make a lasting impact on the lives of those around you and generations to come. We interviewed women in a variety of stages in their career, who have either left a strong legacy behind or are continuing to build their legacy. Each interviewee was asked about the legacy they hope to leave behind, and we found the emergence of two themes: being remembered for their devotion to patients and educating their trainees.

### Perspectives of heart failure cardiologists

As each of these physicians has established themselves as leaders in the field, they understand it is their responsibility to train the next generations of providers. For many of these women, having personally felt the impact of meaningful mentorship in their own careers, training goes beyond a responsibility but is also a passion. One such example of this passion was described by Dr. Maya Guglin, Medical Director of AHF, Heart Transplant and MCS Services at Indiana University and editor of The VAD Journal. The transition from practicing medicine in the Soviet Union to the United States had many challenges, but it was the belief and efforts of her mentors that helped her persevere and start on a path as a leader and an educator. Dr. Guglin experienced first-hand the difference a quality mentorship can make to a career and has made an effort to continue such support and encouragement. She hopes to leave a lasting legacy as an educator. “You leave part of your soul, part of your personality, part of your knowledge in the people you train.” Many of the other physicians we spoke to aligned with Dr. Guglin’s sentiments. They enjoy seeing the growth and ownership of those they have mentored.

These women gave a wide variety of advice to mentees, but the overarching theme was to follow their heart and pursue the passion. The work requires long hours which are physically and emotionally draining, but then love for your work will carry you through those hard days. One profound piece of advice was given by Dr. Jennifer Cowger, Medical Director of the MCS Program and Co-Director of the Cardiac Critical Care Unit at Henry Ford Health. She advised aspiring AHF and transplant specialists to define their careers and craft their path. In her words “your institution doesn’t make your career; you make your career.” This encompasses the grit and perseverance required of so many of these women throughout their careers. Many faced struggles—both gender and non-gender based—but in order to establish themselves as leaders of their field they leaned on their confidence in their abilities. The determination and grit put forth by these women, and so many more trailblazing women in the field, has left an inspiring path for future providers of AHF patients.

### Perspectives of cardiothoracic surgeons

Continued education is a strong component of many CT surgeons’ backgrounds due to the constantly evolving technologies of MCS and transplant. Some of the women we interviewed took this background a step farther and made it a priority in their mentorship. Understanding education can be more than just teaching new techniques and technologies but also inspiring youth to pursue such fields or establishing stronger patient-care cultures. One example of a surgeon who has worked to leave behind a legacy as a passionate educator is Dr. Margarita Camacho, who feels most proud of her work educating future generations. This passion ranges from training CT fellows to currently doing bi-monthly lectures for high school students at her local science center. Though education is a point of great pride for Dr. Camacho and so many other surgeons we spoke to, they derive most satisfaction from the positive patient experiences and outcomes and define their legacies. Dr. Camacho spoke about the joy she experiences seeing a transplant recipient doing well after surgery and it is this joy that can help get one through tough situations. She spoke of one of her mentors, thoracic surgeon Dr. Carolyn E. Reed, who kept thank you cards from patients in a shoe box. Dr. Reed would go to that shoe box to help her whenever she experienced cases with more emotionally taxing outcomes. Many of the surgeons we interviewed discussed how they wish to be known for their patient care.

The desire to leave a legacy of being a patient-centered physician was also an important component in their motivations to choose the field. One piece of advice was to ensure one truly has a passion for the work. As all the surgeons we interviewed discussed, CT surgery is incredibly demanding and can be very tough to balance with one’s personal life. One needs grit, perseverance, and exceptional time management skills to succeed, but most importantly one needs passion for work to sustain in the field. As Dr. Camacho said, “passion will get you through the down days and make the good days exhilarating.” Other surgeons agreed and felt it was important to think critically about how CT surgery fits into one’s personal life in order to find balance and remain as mentally, emotionally, and physically healthy as possible. The surgeons we interviewed, and many more not in this piece, have prioritized others—their patients and mentees—to better clinical outcomes and further the abilities of future generations of surgeons.

## Discussion

A medical school campus may not reflect gender imbalance in today’s day and age; however, it is very apparent in clinical practice, societal conferences, and leadership summits (17). Despite unlocking the door and achieving tremendous progress over the past century, women continue to be underrepresented in traditionally male dominated fields such as CT surgery and cardiology at all levels with stark gaps in senior academic ranks, leadership, and executive positions. This issue is not isolated to the United States, as the literature reports similar gender-based discrepancies in Canada and across Europe (18, 19). While the magnitude of the gap may vary between the countries and continents, disparities based on gender and vertical segregation of women in cardiology and CT surgery are pervasive across the globe. Borelli et al. performed a gender-based analysis of the distribution of leadership positions within cardiology departments in a cohort of 23 European countries, finding significant disparities similar to the United States (20). Although different perspectives and hypotheses are postulated for these inequities, the underlying reasons are complex and multifold and differ a great deal depending on the domain of practice, type of practice and level of career. Cultural stereotypes and social expectations of women’s role in family and society plays a role in these disparities. Some suppose that women tend to voluntarily drift away from the specialties that are more demanding due to their preference of wanting more flexibility to balance their domestic responsibilities, childcare needs, or caregiver responsibilities. Though this may be true in some women, it is unlikely to be the overarching reason. Heart failure field, despite being known for high intensity, acuity, and demanding work hours, attracts more women in cardiology than other procedure based subspecialties such as electrophysiology and interventional cardiology dispelling the myth of desire for flexibility as the reason for deterrence.

**FIGURE 2:**
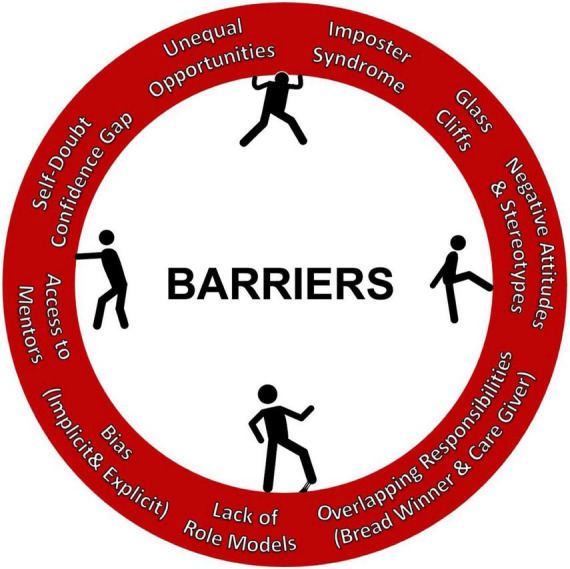
Barriers to advancement for women in CT surgery and heart failure specialist.

**FIGURE 3:**
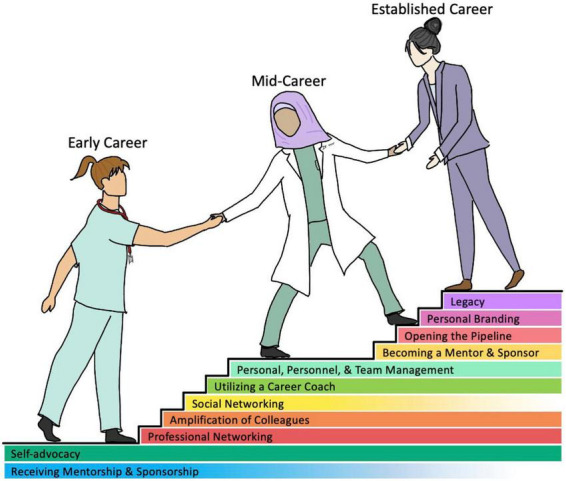
Strategies for aspiring and current women of heart failure and CT surgery can utilize to persevere through gender-based challenges.

A systematic review of heart failure clinical trials by Whitelaw et al. revealed that women were underrepresented as authors without any significant change in the representation patterns in the past two decades. Based on randomized controlled trials studying heart failure published from 2000 to 2019 in high impact journals, women were represented in meager portions as lead (15.6%), senior (12.9%) and corresponding authorship (11.4%) roles (21). Homophily in the professional and societal circuits, lack of investment in diversity and inclusion, and lack of or limited institutional support and sponsorship from professional organizations and industry can undermine the professional development of women and perpetuates the entrapment of the sticky floor and glass ceiling. Nonetheless, losing a deserving, talented and high caliber physician for these reasons is a disservice to medicine, patients, and society. Investment in human capital to reduce the gender gap by addressing the barriers for entry and growth in these fields is essential to achieve gender equality and promote diversity, Figure 2. In contrast to male physicians and surgeons, the trajectories of women entering these rigorous fields differ greatly due to the meager presence of women mentors and role models as men have been at the center stages of these fields for decades. This leaves women in medicine less prepared to navigate the work environment in clinical practice and academic hierarchy as well as integrating into the fabric of professional societies. Several factors including implicit and explicit biases, attitudes and traditional norms pose challenges for women in traversing the crucial organizational structures to ascend in their careers, climb the leadership ladder, attain senior ranking positions, and grow in organized medicine. A complex interplay of several of these factors make women more susceptible to imposter syndrome. Acknowledging the existence of an imbalance and understanding its etiology is complex, we must then ask: how do we move forward? The responsibility of leveling the field of AHF not only falls on aspiring women physicians and surgeons but is also in the hands of members of all genders, generations, and geographies. It is essential for the institutions and organizations to define operating models for partnerships to advance principles of equality, diversity and inclusion and promote professional development of women in this field, Table 3. Although no strategy is exclusive to any career level, we broadly categorized strategies for women to advance their professional growth and presented a model by career stages as represented in Figure 3.

**TABLE 3:** Strategies to improve representation and professional advancement.

Individual	Institutional and organizational	Professional societies and editorial boards	Policy and oversight
Seeking mentors, sponsors, and allies	Incentivize and promote diversity in recruitment and foster inclusive culture	Promote and facilitate diversity and gender balance in societal activities	Policy and legal frameworks to support inclusive and equitable culture
Amplification through networking and social media	Programs to identify, acknowledge and address negative attitudes and stereotypes	Diverse and gender balanced panels and editorial boards	Incentivize diversity and recruitment and promotion
Utilizing digital and social media tools for peer promotion and self-promotion	Establish women leadership councils (WLC) and women resource groups (WRG)	Engage women and active sponsorship of women	Promote gender diversity in leadership ranks
Seeking education and training programs to navigate identity shifts with career advancement	Institutional training programs to increase the awareness and training on: - gender stereotyping - conscious and unconscious biases	Establish and sponsor women sections	Establish policies to promote gender neutral compensation structures and close gender-based wage gap
Seizing opportunities	Leadership development and career advancement programs geared toward women	Scholarships and coaching programs for women	Mandate salary equity reviews
Building peer support community and strategic partnerships	Structured programs and pathways for mentoring women		
Acknowledging and addressing feelings of “Impostorism”	Promote work culture to facilitate work-life balance and/or Breadwinner-caregiver responsibilities		
Resilience training	Active sponsorship of women and build diverse pipeline		
Engaging with women sections in professional societies and organizations			
Gaining agency			

For women early in their career, mentorship is critical to get their foot in the door. While gender concordance is not an essential element in mentorship, it is of value to have relatability when seeking advice on gender specific challenges. While the benefits of mentorship are vast and undeniable, it is not sufficient to achieve equality. One size does not fit all, and women need advocates, coaches, allies, and sponsors to aid in professional advancement. Amplification is one of the methods that can be utilized to promote the work of women in their early careers. The power of social media can be harnessed to promote crowdsourcing in a goal directed manner for this purpose as reflected in the social media campaigns #NYerORCover Challenge launched by Dr. Susan Pitt and #ILookLikeASurgeon set in motion by Dr. Heather Logghe (22). The connectivity supplied by social media can also promote opportunities for FIT and/or early career women to join peer support groups or engage in a wider range of professional networking (23, 24). Such methods of encouragement and inclusivity are also being practiced outside of the United States. Pompili et al. highlight the efforts made by the European thoracic surgery community to develop mentorship programs as well as female surgical associations and conferences (25). In addition, organization such as the Pink International Young Academy of Cardiology provides support and mentorship for aspiring female cardiologists internationally (26).

Mid-career women often face the sticky floor concept, where obstacles arise with ascending to higher levels of leadership. Though institutional and organizational cultures play a role in this, women tend to have a confidence gap and question their abilities and underestimate themselves. A change in the attitudes and actions of leaders are warranted within both specialties—of all genders—, our interviews elicited some strategies women may apply. Seeking the advice and guidance of a career coach can empower women to minimize salary gaps, negotiate better contracts for work-life integration, and offer strategies to build their leadership skills. In addition, professional development courses and workshops, leadership courses and certifications and creating networking opportunities will help acquire skill sets and sharpen their approach to professional growth. Creating a diverse and just culture that allows the physicians to reach their best potential and facilitating an environment that allows recruitment and retention of best physicians regardless of sex is critical for success of any healthcare organization.

Women with established careers are likely to have the confidence and fortitude to step outside of the box and exercise their leadership skills and influence the attitudes and practices of their institutions. Institutional and organizational legacy is infested with sexism, racism, and elitism, promoting inequality. This places women at a disadvantage as they are prone to second-generation gender bias and prevents them from securing higher ranks and C-suite positions, keeping the proverbial glass ceiling intact. Women in senior positions often face “double-bind” and often need to shift their choice of words, salary gap despite their accomplishments and struggle with imposter syndrome. Confidence, finding allies, building alliances with key personnel at institutions and organizations as well as creating a brand of their own can help steer their careers and help build a legacy they want to build. Most importantly, they can build pipelines and empower other women. Advocating for a culture of inclusivity that is based off potential and not old notions of gender or racial stereotypes can enhance diversity and a shift in culture of the organization.

Women in these fields continue to swim against the tide to reach their goals and continue to move the needle despite the constraining forces. However, the leaky pipe—a metaphor for the dwindling number of female HF cardiologists and CT surgeons as they progress through their field—must be flushed for a harmonious and healthy landscape. While it can be argued that it is a voluntary choice of women to join the branches of medicine that offer more flexibility and drift away from branches that hinder the work-life balance, it is important to critically analyze why women who are ambitious enough to pursue medical school and training are choosing not to do so in their careers.

### Limitations

This article is generated based on review of information via web search and interviews. The accuracy of the information depends on the veracity of representation of the information on web-based platforms. Unintentional lapses in conceptual understanding and interpretation may limit the representation of views expressed by interviewees.

## Conclusion

The fields of AHF, MCS, and Transplant are truly unique callings. The long hours, difficult conversations, and high acuity of cases in both medicine and surgery demand a true passion for the field and the work. Though the heart failure specialty has a stronger history of including women relative to cardiothoracic surgery, both experience underrepresentation. Countless female heart failure specialists and CT surgeons have persevered for the betterment of younger generations of physicians and their patients. As described by Dr. Manreet Kanwar—current Medical Director of the Cardiac Transplant and MCS Program and co-director of the Division of Heart Failure and Pulmonary Hypertension at the Cardiovascular Institute at Allegheny Health Network—the equities some women are privileged to today comes from standing on the shoulders of Giants who changed the status quo and paved path for future generations. Women face a higher bar for acceptance due to stereotyping, implicit and explicit biases and may be subjected to extra scrutiny and judgment in establishing their validity in the workspace. Gender mainstreaming by taking gender perspectives into account with the goal to achieve gender equality in dialog, advocacy, resource allocation, planning and policy development is central to achieving gender equality. Leveling the field is a responsibility of all and purposeful and deliberate actions by individuals, institutions and organizations are required to reset these deeply ingrained norms and attitudes. Reengineering of organizational frameworks and policies are essential to build complementary support system to empower and promote women along with collective efforts to shift culture, practices and facilitate an equitable pipeline to achieve desired demographic shifts in these conventionally male dominated spheres of medicine and surgery at the highest ranks including leadership and C-suite positions.

## Author contributions

MM involved in conceptualization, project administration, and supervision. KB and MM involved in the planning, execution, writing, and critical appraisal of this manuscript. FK involved in writing and critical appraisal of this manuscript. All authors contributed to the article and approved the submitted version.

## Glossary

**Imposter syndrome**: an internal belief that one is not as competent as others how one is perceived externally.

**Second generation gender bias**: practices that are applied to all genders, therefore do not overtly appear sexist, but promote the values of the dominating gender.

**Double bind**: in gender bias, occurs when two opposing standards are placed on an individual, but both exist with negative connotations (e.g., a collaborative woman is seen as weak and non-assertive, but an assertive woman is seen as aggressive).
